# Supplementary material for: Combined TIRF and 3D Super-Resolution Microscopy for Nanoscopic Characterization of Adhesion Molecules on Microvilli
Source: Anal Chem. 2026 Jun 5;98(23):16898–911. doi: 10.1021/acs.analchem.5c08159 (PMC13276846; doi:10.1021/acs.analchem.5c08159)
Supplement: Supplementary file 1 [file ac5c08159_si_001.zip › Computational Workflow.docx]

**Overview of the Computational Workflow To Integrate Single-molecule Coordinates With 3D Topographic Maps. (Scripts 1–7)**

The Scripts file contains a series of MATLAB and Python scripts that, together, form a complete data-processing pipeline used to extract microvilli contours and associated single-molecule coordinates, reconstruct the TIRFM-based 3D topography and 3D-SMLM intropolated fitted surface, map individual 3D single-molecule localizations to specific microvilli, generating microvilli-specific molecular datasets, and analyze the nanoscale spatial organization of adhesion molecules on microvilli from combined TIRFM/3D-SMLM imaging experiments. This structured computational workflow mirrors the experimental logic described in the manuscript (Figures 3-6 and Supporting Figures S7–S12 and S14-S20), ensuring an accurate and quantitative correlation between cell surface topography and adhesion molecule localization at nanometer-scale resolution.

**Script 1 (Cell3D.m). Reconstruction of the 3D TIRFM-Based Cell Surface Topography and Generation of Microvilli Masks**

Script 1 performs the preprocessing and reconstruction of the cell surface architecture using TIRFM intensity decay and LoG filtering. It also extracts 3D single-molecule positions within the TIRF penetration depth and generates a binary mask representing membrane protrusions (microvilli).

Key Processing Steps:

1. Imports the TIRF image (e.g., 120nm.tif) and 3D SMLM coordinates file (XYZ.txt).
2. Apply a Laplacian-of-Gaussian (LoG) filter to enhance the edges of microvilli and suppress background noise.
3. Convert TIRF intensity values to axial position by implementing the exponential evanescent-field decay model using the selected penetration depth (120 nm).
4. Produce an estimated height map (δz) corresponding to the local membrane–glass distance.
5. Create a 3D mesh surface plot (Xa, Ya, Za) representing the reconstructed 3D membrane topography and save it as TIRF_data.mat.
6. Converts SMLM Z-coordinates to nanometers and selects molecules within the 120 nm penetration depth of the evanescent field (f1–f2) and stores the result in coord.txt.
7. Generate a binary image (binary_image.png) of isolated microvilli based on intensity thresholding (≥200 counts), used later to assign 3D-localized molecules to their respective microvilli.

This script implements the workflow described in Figure 3a-e, which includes LoG filtering, intensity-to-distance conversion, and microvilli contour extraction.

**Script 2 (TIRFanalysis.py). Classification of Microvilli Orientation Using Binary Mask**

Using the binary microvillar mask from Script 1, Script 2 analyzes the binary mask (microvilli contours) and identifies vertical microvilli based on their 2D shape characteristics.

Key Processing Steps:

1. Load the TIRF-derived binary microvilli image (binary_image.png) and detect microvilli contours.
2. Compute the bounding box for each microvilli and classify microvilli as vertical microvilli with near-circular shape (aspect ratio ~1.0).
3. Generate a filtered binary mask containing only vertical microvilli, saved as Vertical_binary_image.png.
4. Visualize the contour classifications for quality control.

This script corresponds to the distinction between vertical and horizontal microvilli and their impact on reconstruction accuracy described in the SSIM analysis shown in Figures 3f, 3g, 4c, and S9. Classification improves the interpretation of the spatial overlap between SMLM localizations and TIRFM-derived topography.

**Script 3 (Filtration.py). Assignment of 3D SMLM Localizations to Individual Microvilli**

Script 3 integrates the microvilli segmentations with the 3D SMLM coordinates to determine which molecules belong to which microvilli. The script performs this twice: first for vertically oriented microvilli alone, then for all microvilli.

Key Processing Steps:

**(A) Vertical Microvilli Only**

1. Load Vertical_binary_image.png and find all microvillar contours.
2. For each contour, generate a filled mask and determine which SMLM molecules, whose (x, y) positions fall within that region, are assigned to that microvilli.
3. Save one output file per microvillus (e.g., Vertical_coordinates/1.txt).
4. Remove empty files and visualize assigned molecules.

**(B) All Microvilli**

1. Repeat the same process using binary_image.png.
2. Generate microvilli_coordinates folder, containing per-microvilli coordinate maps.
3. Save all_filtered_coordinates.txt, representing the union of all assigned molecules.

This script implements the molecule-to-microvillus mapping used in Figures 4c-d. It provides the basis for quantifying the molecular distribution across microvillar structures.

**Script 4 (Draw_filtered.m). Overlay of TIRFM-Derived Topography With Molecules**

Script 4 overlays and visualizes the global agreement between the reconstructed TIRF topography and the spatial distribution of all SMLM detections associated with microvilli.

Key Processing Steps:

1. Load the full molecule dataset (all_filtered_coordinates.txt).
2. Load TIRF_data.mat and render the 3D surface topography.
3. Scatter-plot the assigned SMLM coordinates on top of the surface.

This script operationalizes the pipeline described in previous scripts, where molecules are filtered by axial position, mapped to microvilli contours, and visualized overlaid on the reconstructed cell surface. This overlay corresponds to Figures 5a, 5b, S7, and S20, showing merged topography and molecular localization.

**Script 5 (BivariateHistogram.m). 3D distribution of adhesion molecules relative to the 3D Microvilli topography**

Script 5 extracts a small region around a selected microvillus and performs localized quantitative analyses.

Key Processing Steps:

1. Load all_filtered_coordinates.txt and TIRF_data.mat.
2. Define a region-of-interest (ROI) in X-Y space (e.g., [159-167] × [154-161]).
3. Filter the molecular localizations to those within this ROI.
4. Generate:

- XZ and YZ bivariate histograms
- A localized 3D TIRF surface representation
- Single-molecule overlays within the ROI

1. Apply custom axis scaling to convert pixel coordinates to nanometers.

This script produces the bivariate histogram analyses illustrated in Figure 5b, 5c, 5d, 6a-c, 6e-g, 7b-j, S8, S11, S12, S16, S17, and S19 to determine the 3D distribution of adhesion molecules relative to the 3D Microvilli topography.

**Script 6 (SurfaceFitting.m). Local Surface Fitting of Single-Molecule Data**

Script 6 performs linear interpolation across the molecular dataset to fit a continuous local surface and compare it to the TIRF-derived topography.

Key Processing Steps:

1. Load molecule coordinates and TIRF surface.
2. Define a region-of-interest (ROI) in X-Y space (e.g., [159-167] × [154-161]).
3. Create a scattered interpolant of a single-molecule 3D data set.
4. Generate a fitted surface (Xq, Yq, Zq) across the ROI.
5. Overlay the fitted surface with the TIRF surface and SMLM detections.

This script supports the structural comparison analysis described in the Results section and in Supporting Figure 5e, where fitted molecular surfaces are evaluated against TIRF topography.

**Script 7 (SSIManalysis.py). Structural Similarity Index (SSIM) Analysis Between TIRF Topography and Fitted Molecular Surfaces**

Script 7 performs quantitative similarity analysis for each microvillus by comparing the TIRF-derived surface with the SMLM-derived scattered interpolant local surface using SSIM.

Key Processing Steps:

1. Load TIRF height map (Za) and binary microvilli mask.
2. Identify individual microvilli using regionprops.
3. For each microvillus:

- Extract the corresponding TIRF ROI.
- Load its associated molecular coordinates.
- Fit a 3D interpolated surface using griddata.
- Normalize both surfaces and compute the SSIM.

1. Apply SSIM and a similarity threshold (e.g., 0.3) to determine whether molecular and TIRF surfaces match.
2. Print the SSIM distribution, including:

- Mean SSIM
- Percentage of microvilli with SSIM > 0.3

This script implements the quantitative comparison shown in Figures 5f-h, 6d, 6h, S9, and S10, confirming the consistency of SMLM and TIRF-derived structural information.

**Summary Table**

| **Script** | **Function** | **Corresponds to Manuscript** |
| --- | --- | --- |
| **1** | Reconstruct 3D TIRF surface; generate binary microvilli mask; export SMLM coordinates | Fig. 3a-e |
| **2** | Classify microvilli (vertical) via aspect ratio of contour geometry | Fig. 3f, 3g, 4c, S9 |
| **3** | Assign SMLM molecules to individual microvilli (vertical-only and all) | Fig. 4c-d |
| **4** | Overlay of TIRF topography with SMLM points | Fig. 5a, 5b, S7, and S20 |
| **5** | ROI-based quantitative visualization (XZ/YZ histograms and TIRF + SMLM overlay) | Fig. 5b, 5c, 5d, 6a-c, 6e-g, 7b-j, S8, S11, S12, S16, S17, S19 |
| **6** | Surface fitting of molecular coordinates | Fig. 5e |
| **7** | SSIM-based structural comparison | Fig. 5f-h, 6d, 6h, S9, S10 |
