# Supplementary figures and images for: Combined TIRF and 3D Super-Resolution Microscopy for Nanoscopic Characterization of Adhesion Molecules on Microvilli
Source: Anal Chem. 2026 Jun 5;98(23):16898–911. doi: 10.1021/acs.analchem.5c08159 (PMC13276846; doi:10.1021/acs.analchem.5c08159)

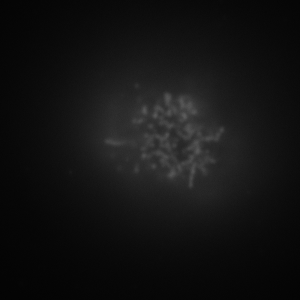

Supplement: Supplementary file 1 [file ac5c08159_si_001.zip › 120nm.tif]
