# Supplementary material for: Combined TIRF and 3D Super-Resolution Microscopy for Nanoscopic Characterization of Adhesion Molecules on Microvilli
Source: Anal Chem. 2026 Jun 5;98(23):16898–911. doi: 10.1021/acs.analchem.5c08159 (PMC13276846; doi:10.1021/acs.analchem.5c08159)
Supplement: Supplementary file 2 [file ac5c08159_si_002.pdf]

Supporting Information

Combined TIRF and 3D Super-Resolution Microscopy for Nanoscopic Characterization of Adhesion Molecules on Microvilli

*Abdullah Alghamdi,[1]† Mansour M. Aldehaiman,[1]† Maged F. Serag,[1] Shuho Nozue,[1] Ioana-Andreea Ciocanaru,[1] Karmen AbuZineh,[1] Jasmeen S. Merzaban,[1] Satoshi Habuchi[1]**

[1] Biological and Environmental Science and Engineering Division, King Abdullah University of Science and Technology, Thuwal 23955-6900, Saudi Arabia.

*Email: satoshi.habuchi@kaust.edu.sa

† These authors contributed equally to this work.

Contents

Supporting Texts 1 to 4

Supporting Figures 1 to 21

Supporting References

Supporting Text

1. Calculation of the critical angle of the total internal reflection (TIR) and the maximum angle in TIRFM

The critical angle at which total internal reflection occurs (θ_{critical}) at the glass/buffer interface was determined using the following equation:

$$\theta_{\text{critical}} = \sin^{-1}\left(\frac{n_1}{n_2}\right) = 61.04°$$

where n_1 = 1.33 (cell cytosol) and n_2 = 1.52 (refractive index of the coverglass and immersion liquid). The maximum achievable TIR angle (θ_{max}), constrained by the numerical aperture (NA) of the TIRF objective lens (NA = 1.49), was calculated using the following equation:

$$\sin\theta_{\text{max}} = \frac{\text{NA}}{n_2} = 78.6°$$

2. Verification of the incident angles and penetration depths of the excitation light

To verify the accuracy of the incident angles of the TIR illumination and, therefore, the penetration depth of the evanescent field, we measured integrated fluorescence intensities under varied TIR illumination conditions (i.e., varied TIR angles). The integrated fluorescence intensity (I) obtained from a solution containing a uniform concentration of fluorophores illuminated at a specific TIR angle (θ) is obtained using equations S1 and S2 and is described as follows:

$$I = I_0\left(\frac{\lambda}{4\pi\sqrt{n_1^2\sin^2\theta - n_2^2}}\right) \quad \text{(S1)}$$

where I_0 is the integrated fluorescence intensity obtained at the critical angle for the TIR illumination, λ is the wavelength of the illuminating light, and n_1 and n_2 denote the refractive indices of the glass coverslip and solution, respectively. We fitted the TIR illumination angle-dependent integrated fluorescence intensity using the following equation:

$$I = I_0\left(\frac{\lambda}{4\pi\sqrt{n_1^2\sin^2\theta - n_2^2}}\right) + bg \quad \text{(S2)}$$

where bg is the background signal on the measured fluorescence images.

3. Accuracy of the TIRF-based topography calibration.

We experimentally measured the integrated fluorescence intensity of a homogeneous fluorophore solution at multiple TIRF incident angles and fitted the resulting data to the theoretical TIRF decay model using two fixed and two free parameters (one of which was the refractive index, n_2). The fitted n_2 value closely matched the actual refractive index of the buffer solution, confirming the validity of our calibration. As shown in Figure S5, the fit closely follows the theoretical curve across most angles, with noticeable deviations appearing only near the critical angle ($61° < \theta < 65°$), a region known to be sensitive to small angular uncertainties. To minimize this source of error, we restricted our topography analysis to angles greater than 65°, where the angle-determination error was minimal and the model fit remained robust.

Importantly, this experimental calibration approach provides more reliable penetration-depth estimation than earlier TIRF-based 3D mapping,[1,2] which fully relied on the theoretical calculation without experimental validation. We also note that a recent study similarly relied on theoretical decay curves without incorporating experimental validation or angle-error assessment.[3]

These methodological optimizations, including experimental calibration, restricted angle range, and error-aware fitting, ensure that the conversion from evanescent field intensity to topographic height is quantitatively reliable within the selected range.

4. SSIM sensitivity simulation

To evaluate the sensitivity of the structural similarity index (SSIM) to variations in molecular localization, we simulated surface datasets with controlled positional displacements of localizations relative to an idealized microvillus surface (Figure S21). Despite large shifts in localization position, SSIM values remained within a narrow range, confirming that the metric primarily reflects overall structural overlap rather than precise spatial alignment.

Supporting Figures

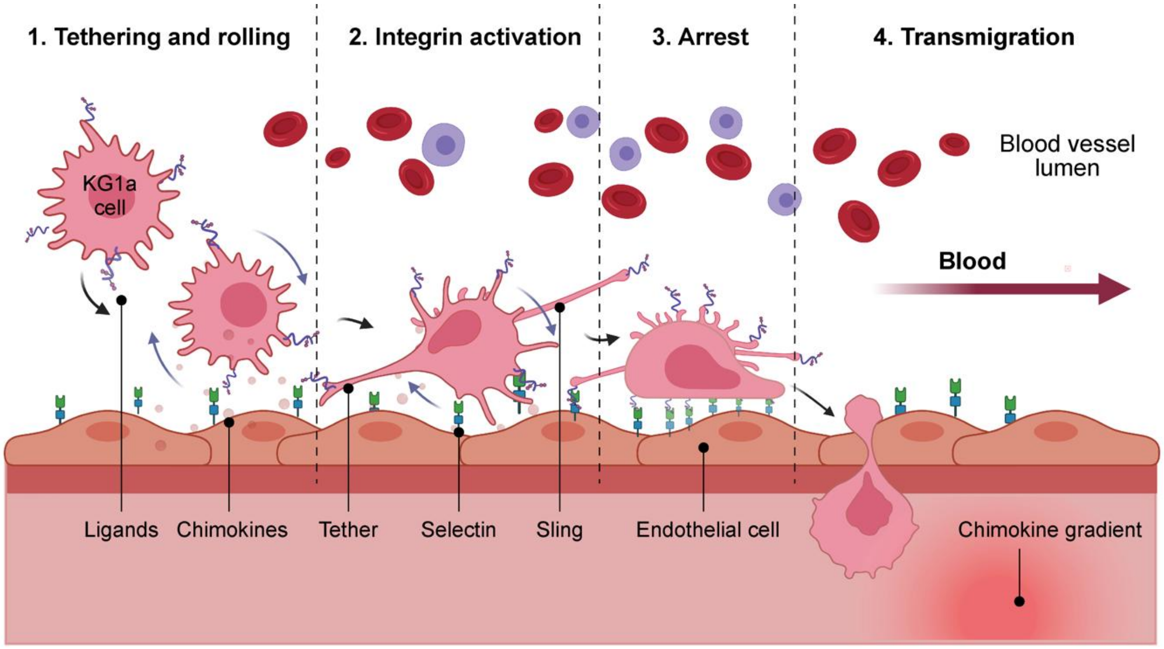

Figure S1. Schematic overview of the hematopoietic stem/progenitor cell (HSPC) homing mechanism. HSPC homing consists of five sequential steps: (1) Tethering and rolling, initiated by the binding of selectins expressed on endothelial cells to selectin ligands on the rolling HSPCs. (2) Activation, triggered by inflammatory signals, leading to cellular responses. (3) Arrest, facilitated by integrin binding, resulting in firm adhesion. (4) Transmigration, where HSPCs migrate through the endothelial layer and basement membrane. (5) Directed migration, where HSPCs move toward chemokine gradients to reach their target niche.

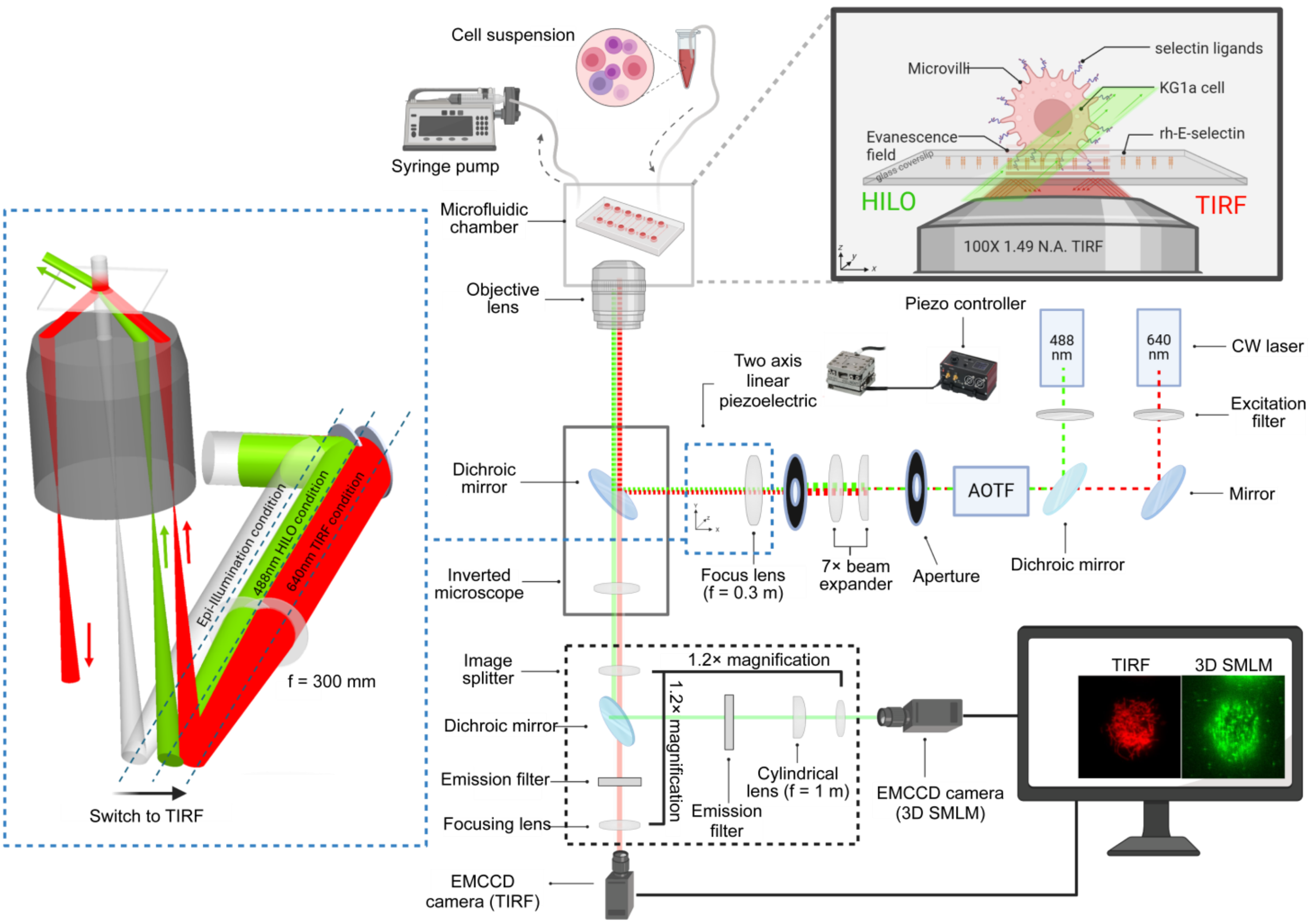

Figure S2. Schematic diagram of a combined TIRFM and 3D-SMLM setup. The setup allows reliable and reversible switching between total internal reflection (TIR) illumination and highly inclined and laminated optical sheet (HILO) illumination. Microfluidic devices required for the cell rolling assay are integrated into the microscope so that the combined TIRFM and 3D-SMLM imaging experiment can be conducted on the rolled cells.

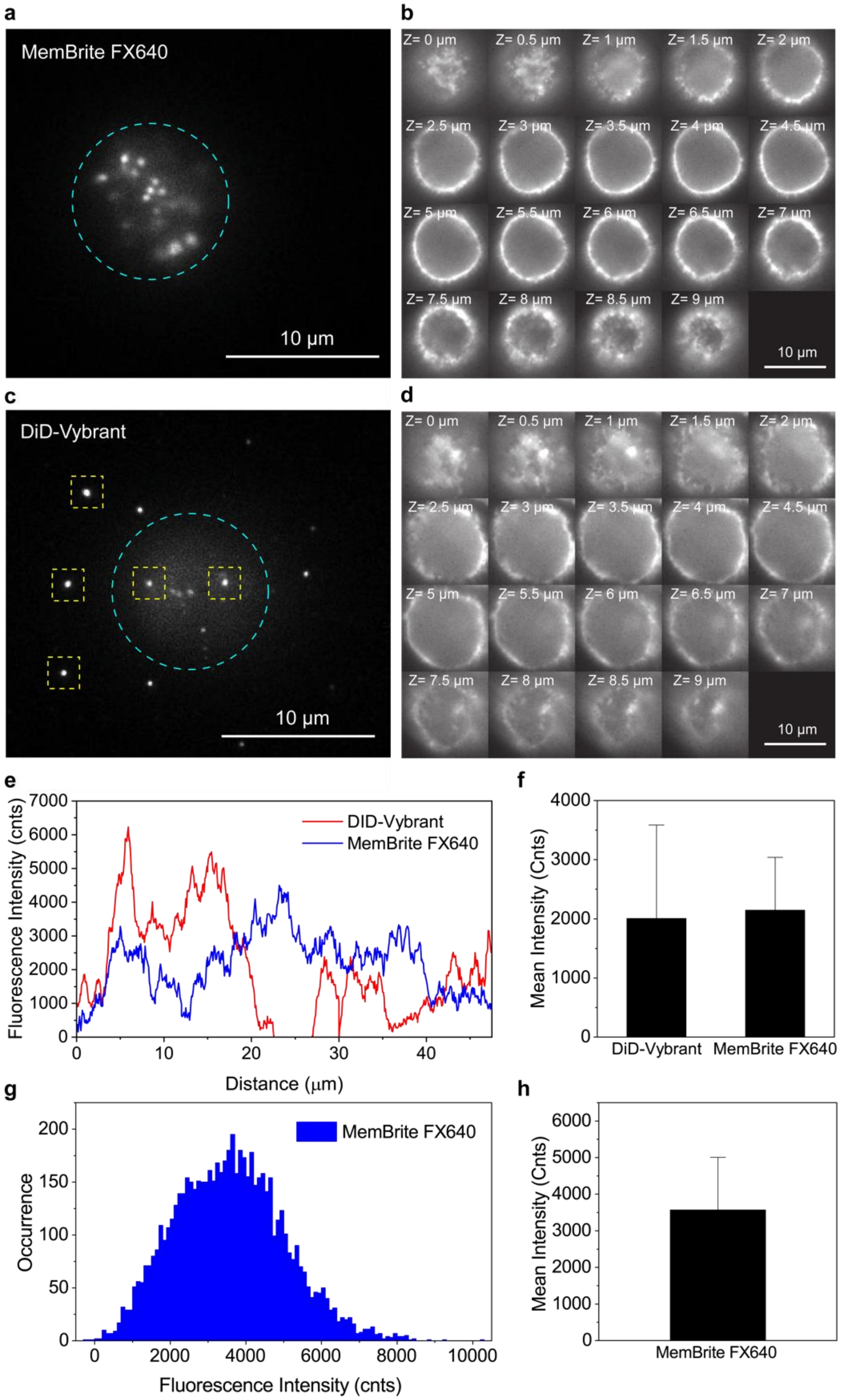

Figure S3. Uniform labeling of the cell membrane using membrane stains. (a) TIRF image of the bottom surface of a KG1a cell stained by MemBrite FX640 stain. The cyan circle indicates the cell

surface region. (b) Fluorescence images of KG1a cells stained with MemBrite FX640 captured at varied Z-axis positions using wide-field epi-illumination fluorescence microscopy. (c) TIRF image of the bottom surface of a KG1a cell stained by DiD-Vybrant stain. The cyan circle indicates the cell surface region. Yellow squares show dye aggregates. (d) Fluorescence images of KG1a cells stained with DiD-Vybrant captured at varied Z-axis positions using wide-field epi-illumination fluorescence microscopy. The Z-axis values represent the distances from the coverslip surface. (e) Analysis of fluorescence intensity variation across the cell membrane at the middle cross-section of the cell in MemBrite FX640– and DiD-Vybrant–stained KG1a cells displayed in (b) and (d). The fluorescence intensity profiles were obtained by averaging the IntDen of 10-pixel regions along a 3 μm length. (f) Mean intensities of the fluorescence intensity profiles in (e). The error bars represent the standard deviations of the intensity profiles. (g) Analysis of fluorescence intensity variation across the cell membrane at the bottom of the cell in MemBrite FX640–stained KG1a cells displayed in (b). Frequency histogram of fluorescence intensity values of each pixel in the image. (h) Mean intensity of the fluorescence intensity distribution in (g). The error bar represents the standard deviation of the intensity profile.

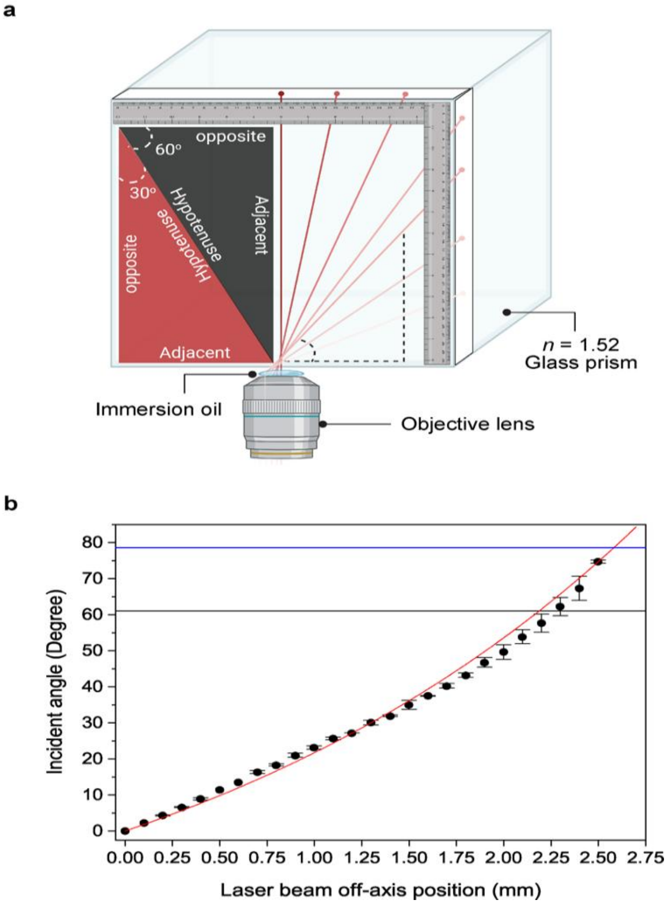

Figure S4. Determination of the incident angles. (a) Schematic diagram of the experimental setup for determining incident angles, where a glass prism with a refractive index of 1.52 is mounted on an inverted microscope stage in direct contact with a TIRF objective lens (N.A. = 1.49). An oil immersion layer was applied between the objective and the prism to match the refractive index. Incident angles were experimentally determined using trigonometric functions. (b) Experimentally determined incident angles at varied laser beam off-axis positions at the back focal plane of the objective lens. The black horizontal line represents the theoretically calculated critical angle for TIR illumination with the TIRF objective lens (61°, see Supporting Text 1). The blue horizontal line represents the maximum TIR angle achievable using the TIRF objective lens (79°, see Supplementary Text 1). The red line shows a single-exponential fitting.

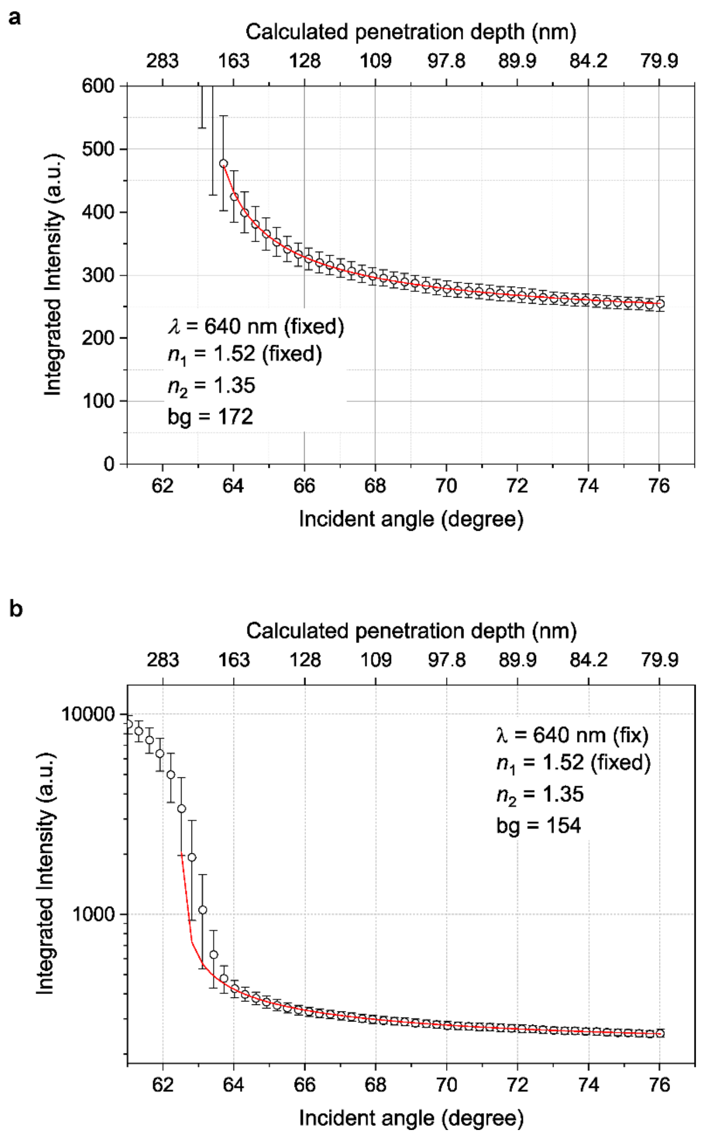

Figure S5. Verification of the incident angles and penetration depths of the excitation light. Mean integrated intensity profile of the 10 μg mL-1 AF640 fluorophores in an aqueous solution, measured across different TIR illumination angles. The error bars show standard deviations of 3 independent experiments. Red lines show fittings to equation S2 (see Supplementary Text 2), where λ is the wavelength of the excitation light, n1 and n2 denote the refractive index of the glass coverslip and the solution, and bg is the background signal in the imaging experiments. The range of the incident angle fitted to equation S2 was (a) 63.7° - 78° and (b) 62.4° - 78°.

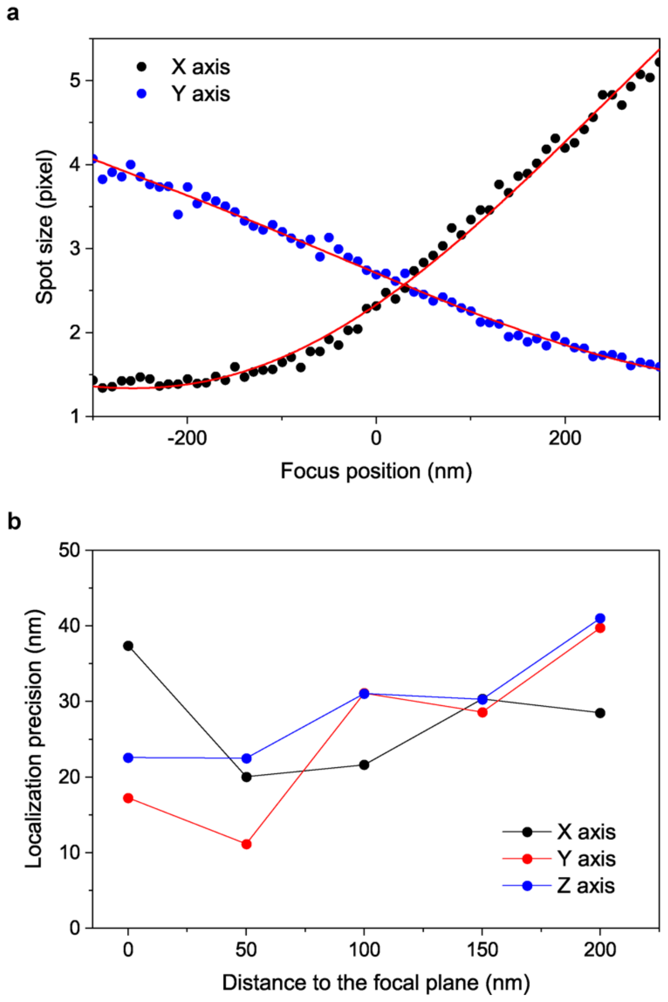

Figure S6. Localization precision of 3D-SMLM. (a) Z-position-dependent widths (standard deviations of the 2D Gaussian used for the fitting) of the fluorescent spots along the X- and Y-axes obtained for TetraSpeck fluorescent nanospheres (100 nm diameter). The red lines show fitting to polynomial functions, which were used for the Z-axis localization of the fluorescent molecules. (b) Localization precisions along X-, Y-, and Z-axes (standard deviations of the XYZ coordinates localized by 3D-SMLM) at varied distances from the focal plane. Localization data were obtained using 20 nm TetraSpeck fluorescent nanospheres captured over 10,000 frames.

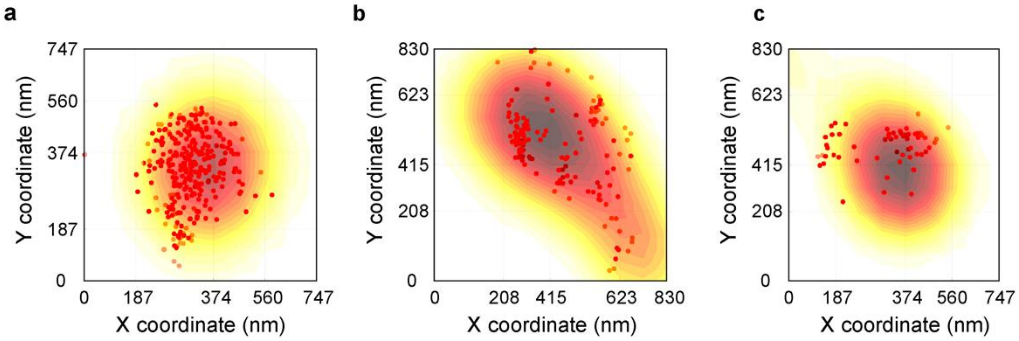

Figure S7. Spatial distribution of adhesion molecules on microvilli projected on the XY plane. XY projections of 3D single molecule localizations of (a) F-actin, (b) CD44, and (c) PSGL-1 on KG1a cells determined by 3D SMLM overlaid with a 3D cell surface topography image acquired by TIRF microscopy with MemBrite FX640 stain. The F-actin, CD44, and PSGL-1 were labeled by AF-488-conjugated phalloidin (F-actin) or immunolabeled with AF-488-conjugated antibodies (CD44 and PSGL-1). (a), (b), and (c) are the XY projections of the 3D images shown in Figure 5b, 6a, and 6e.

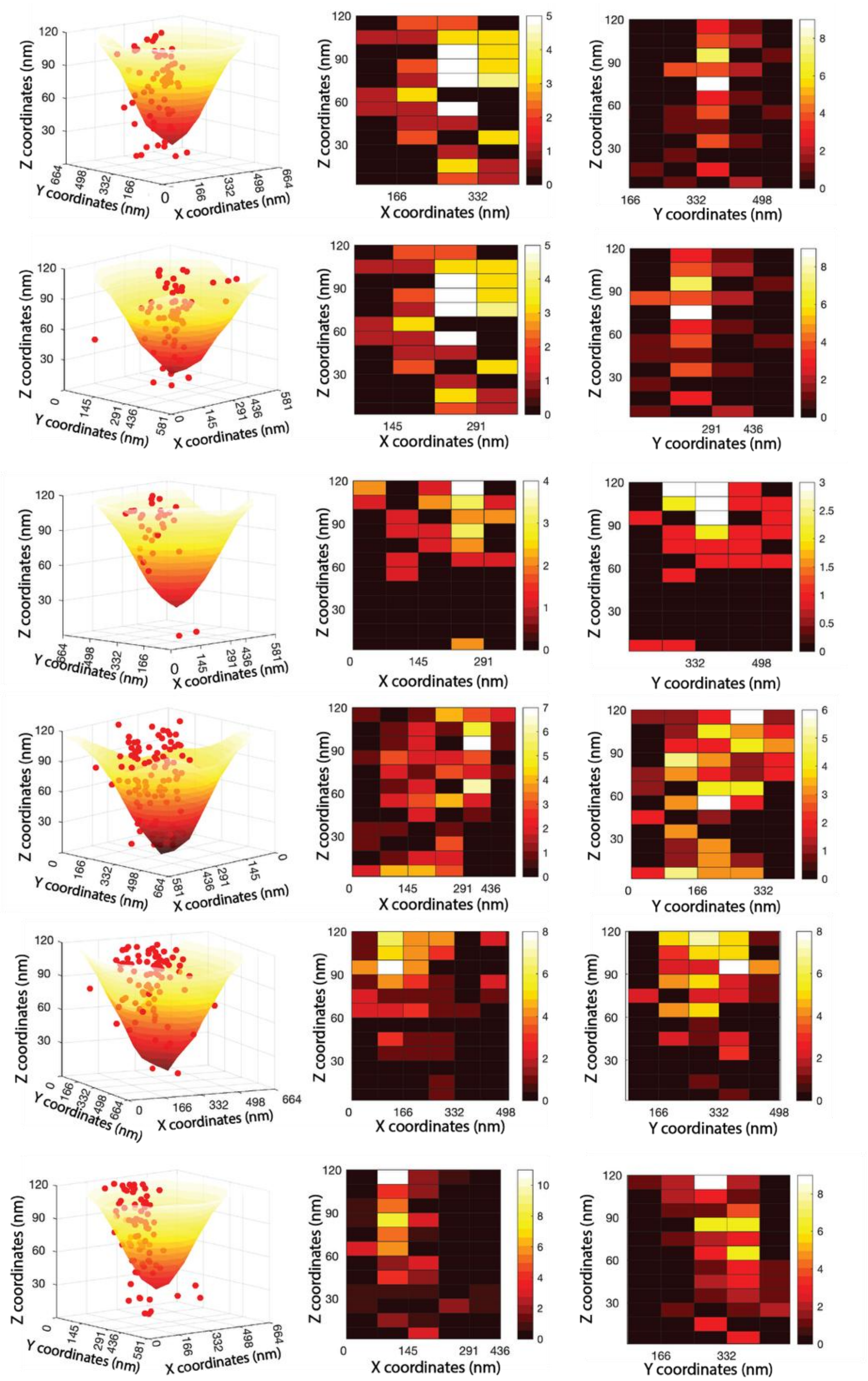

Figure S8. Additional examples of 3D-SMLM localizations of F-actin on vertically oriented microvilli in control KG1a cells.

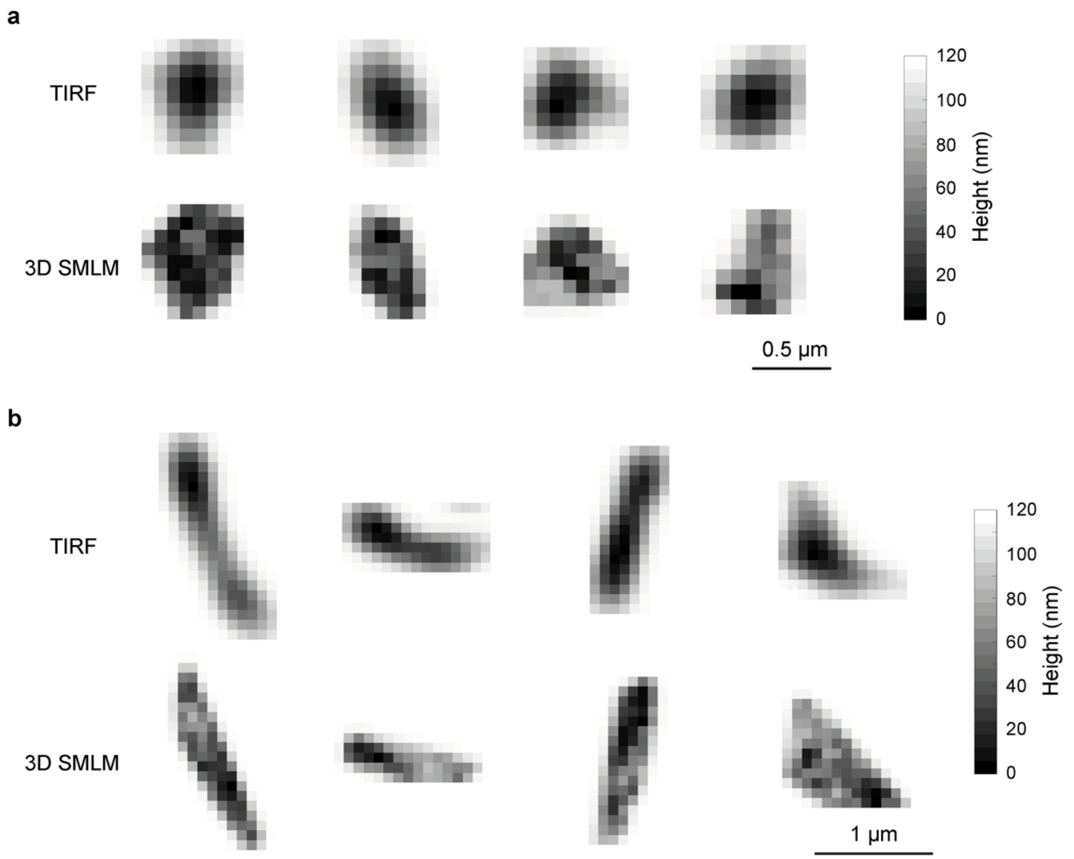

Figure S9. Comparison of the 3D topographic image of the cell surface and the 3D spatial boundary obtained from the 3D coordinates of individual F-actin filaments. Examples of normalized 8-bit 2D images obtained from the 3D topography of a single microvillus (top) and 3D spatial boundary obtained from the 3D coordinates of individual F-actin filaments (bottom). The axial distance from the coverslip surface is shown in grayscale. (a) Vertically oriented and (b) horizontally oriented microvilli were captured in the imaging experiment.

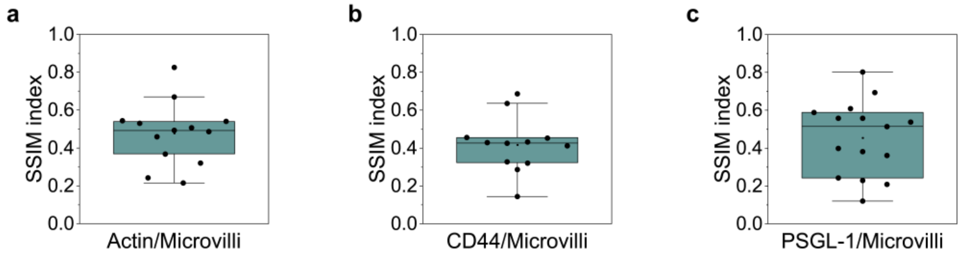

Figure S10. Structural similarity (SSIM) index analysis of the 3D topography of the microvilli generated by TIRFM and the 3D coordinates of the (a) F-actin, (b) CD44, and (c) PSGL-1 molecules determined by 3D-SMLM.

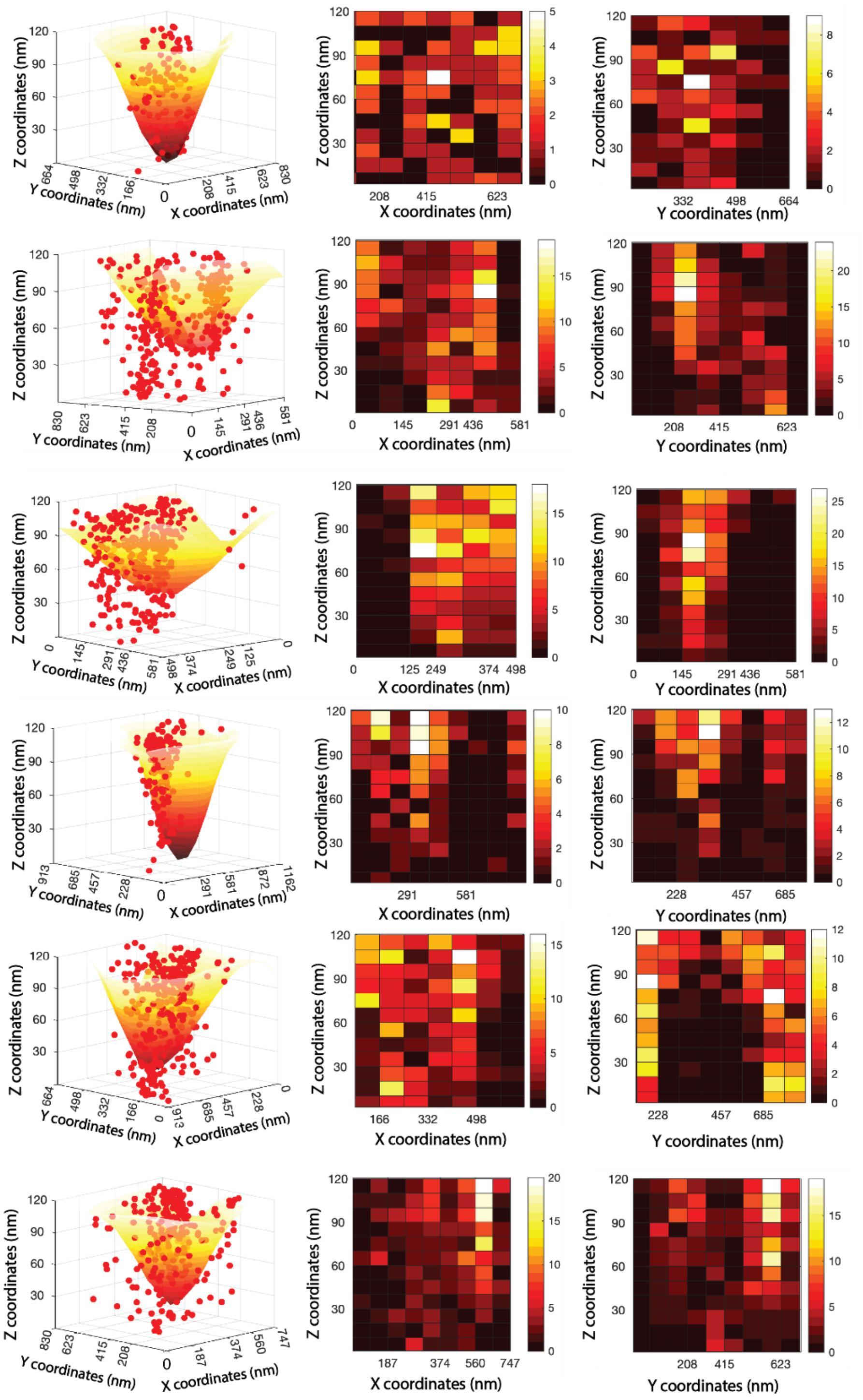

Figure S11. Additional examples of 3D-SMLM localizations of CD44 on vertically oriented microvilli in control KG1a cells.

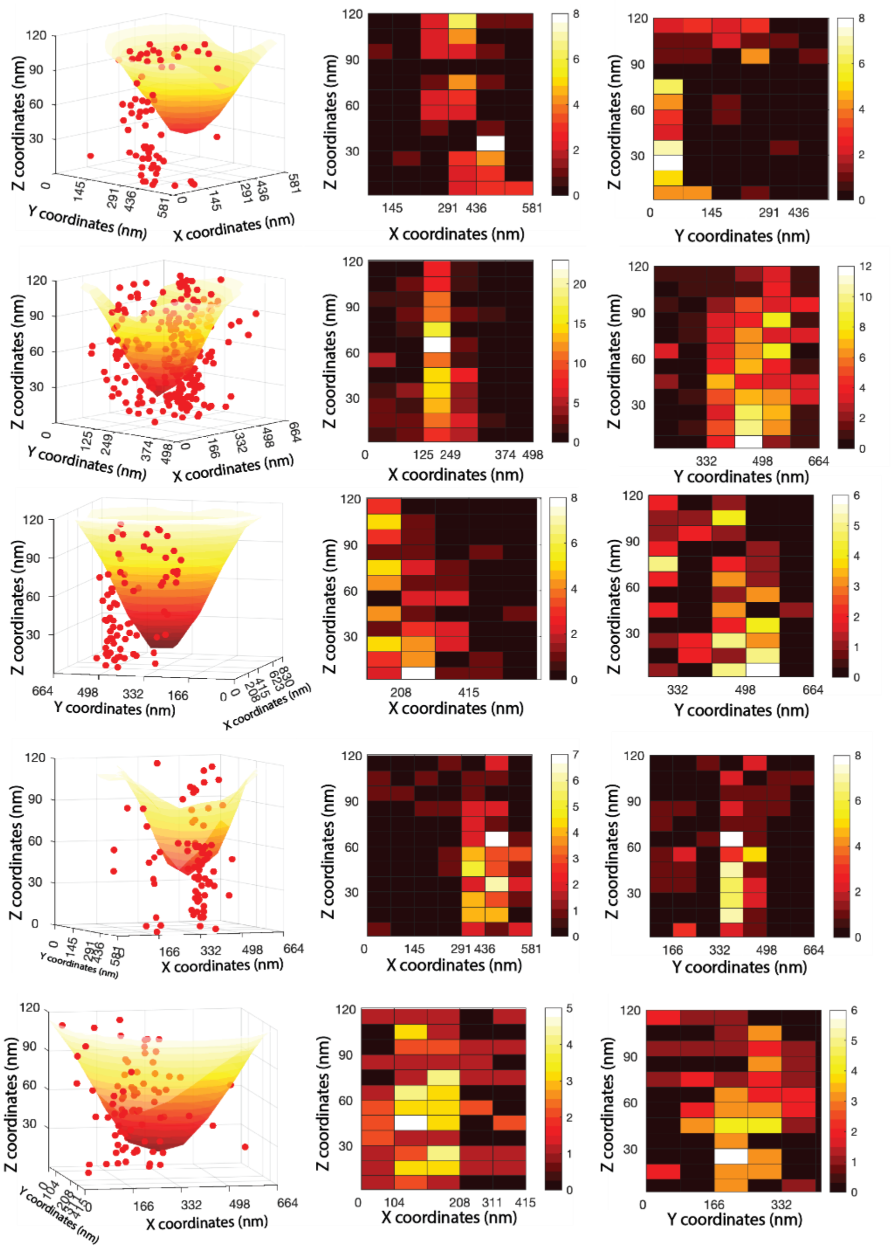

Figure S12. Additional examples of 3D-SMLM localizations of PSGL-1 on vertically oriented microvilli in control KG1a cells

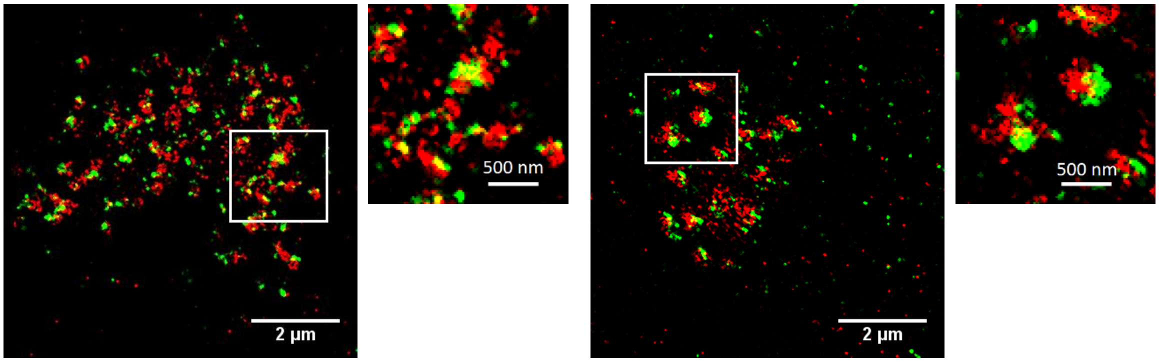

Figure S13. Examples of the two-color single-molecule localization microscopy imaging of CD44 (red) and PSGL-1 (green) on KG1a cells (left). The cells were fixed and immunolabeled for CD44 and PSGL-1 using the IM7 antibodies conjugated to AF-647 dyes and the KPL-1 antibodies followed by AF-488-conjugated secondary antibodies, respectively. The cells were fixed and immunolabeled in suspension. The right panels show enlarged views of the white rectangle regions.

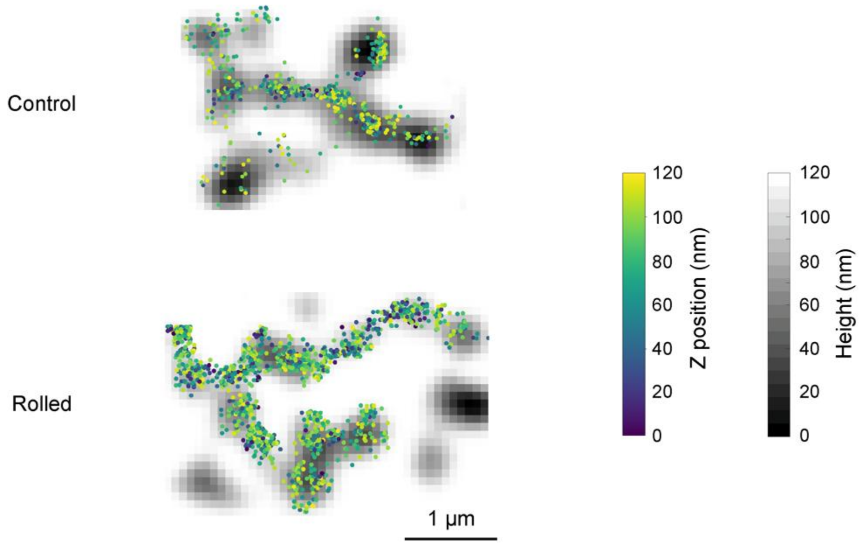

Figure S14. Effect of cell rolling on the architecture of microvilli and spatial distribution of phalloidin-labeled F-actin. The grayscale cell surface topography images obtained by TIRFM are overlaid with the 3D coordinates of actin determined by 3D-SMLM. The grayscale shows the distance from the coverslip surface, and the color scale represents the Z-position of the actin filaments. The top and bottom panels show examples obtained for control and rolled cells over E-selectin.

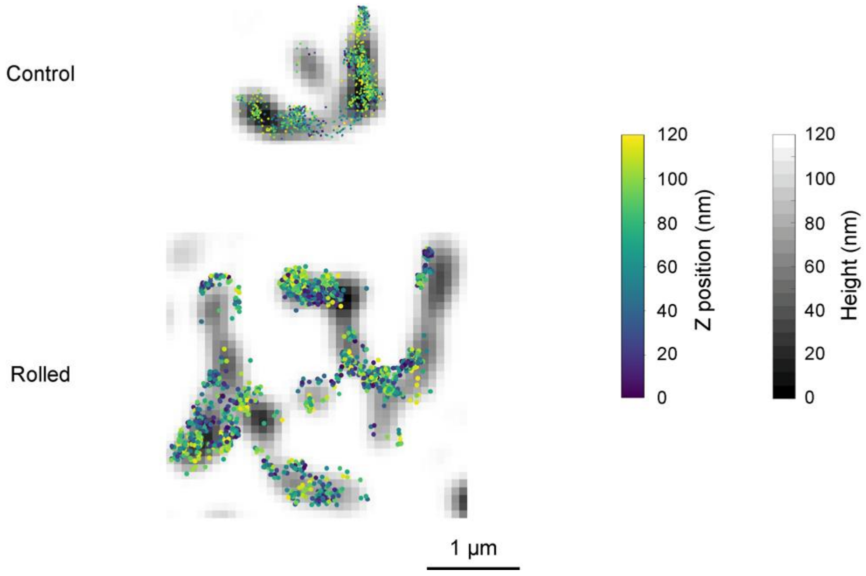

Figure S15. Effect of cell rolling on the architecture of microvilli and spatial distribution of CD44. The grayscale cell surface topography images obtained by TIRFM are overlaid with the 3D coordinates of CD44 determined by 3D-SMLM. The grayscale shows the distance from the coverslip surface, and the color scale represents the Z-position of the CD44 molecules. The top and bottom panels show examples obtained for control and rolled cells over E-selectin.

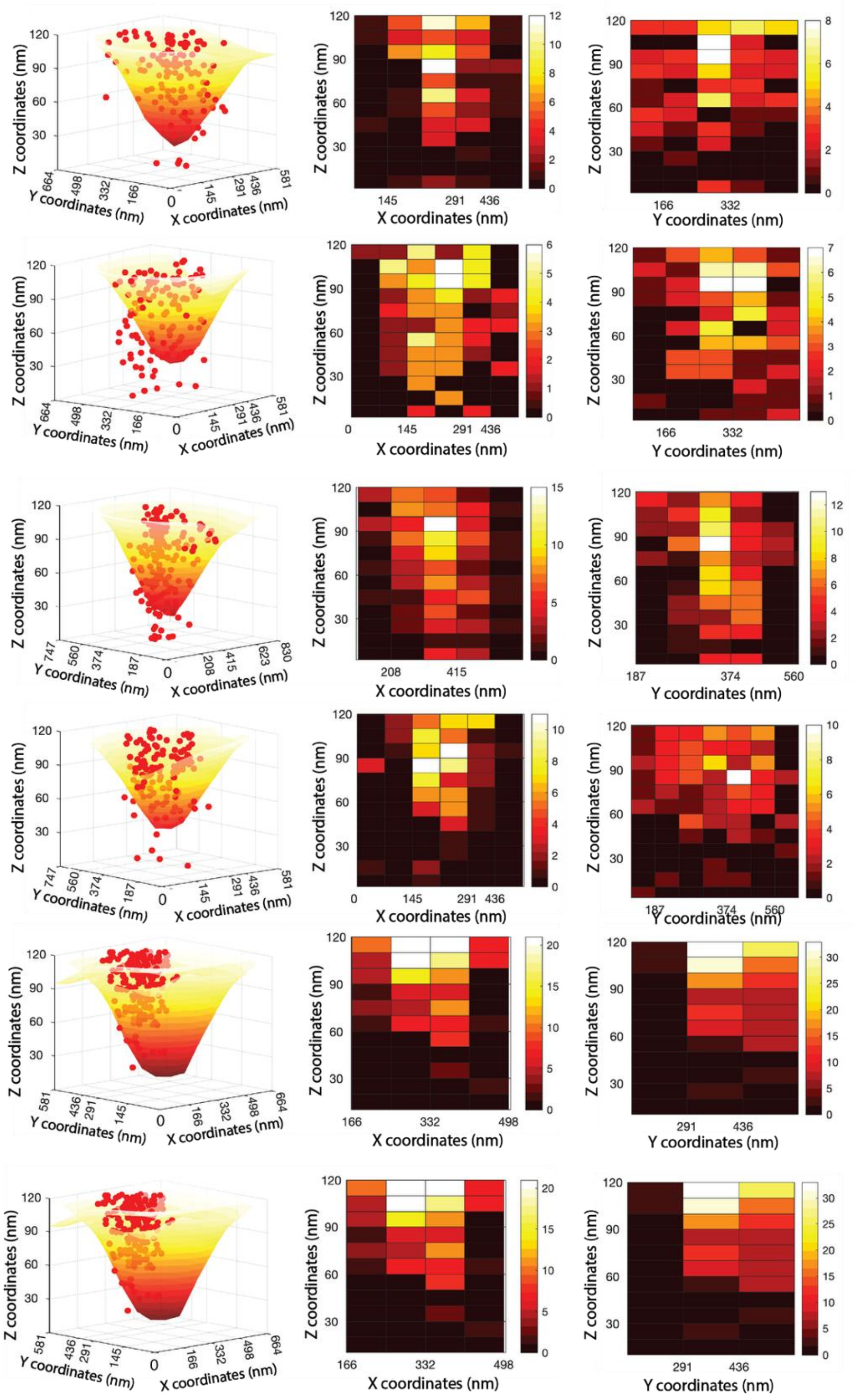

Figure S16. Additional examples of 3D-SMLM localizations of F-actin on vertically oriented microvilli in KG1a cells under shear flow.

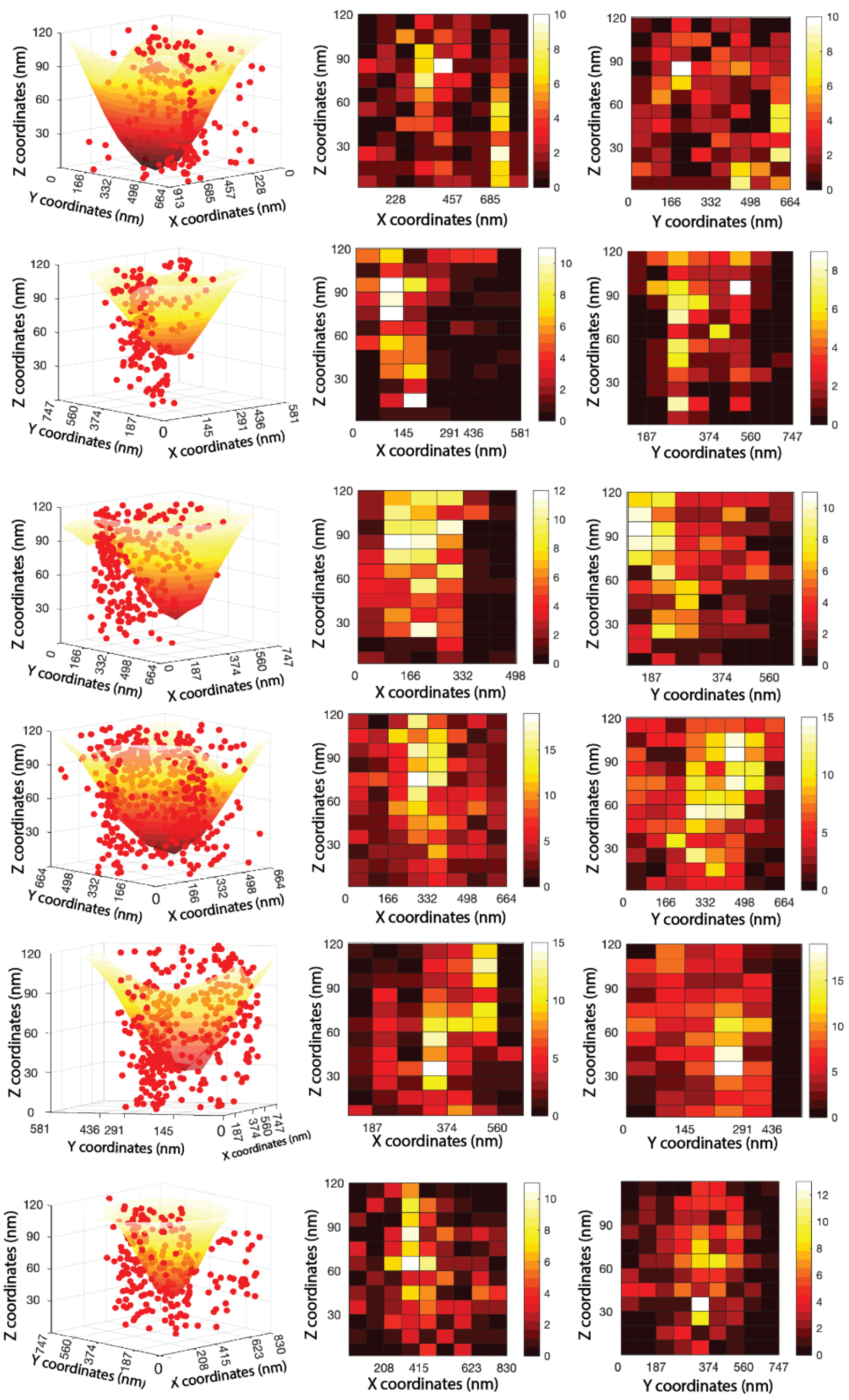

Figure S17. Additional examples of 3D-SMLM localizations of CD44 on vertically oriented microvilli in KG1a cells under shear flow.

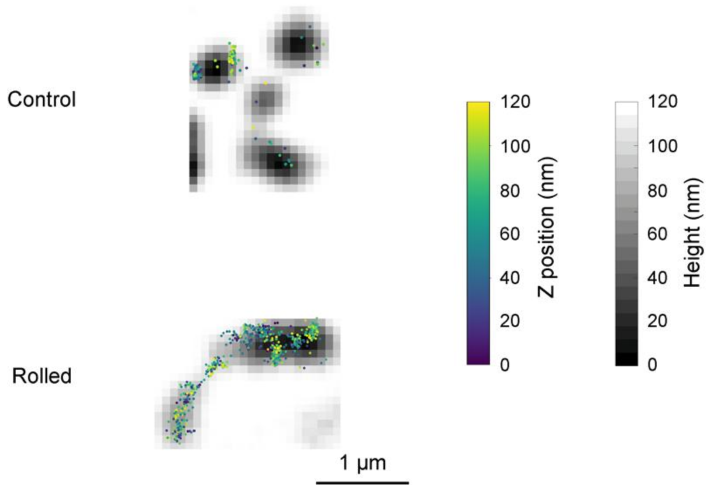

Figure S18. Effect of cell rolling on the architecture of microvilli and spatial distribution of PSGL-1. The grayscale cell surface topography images obtained by TIRFM are overlaid with the 3D coordinates of PSGL-1 determined by 3D-SMLM. The grayscale shows the distance from the coverslip surface, and the color scale represents the Z-position of the PSGL-1 molecules. The top and bottom panels show examples obtained for control and rolled cells over E-selectin.

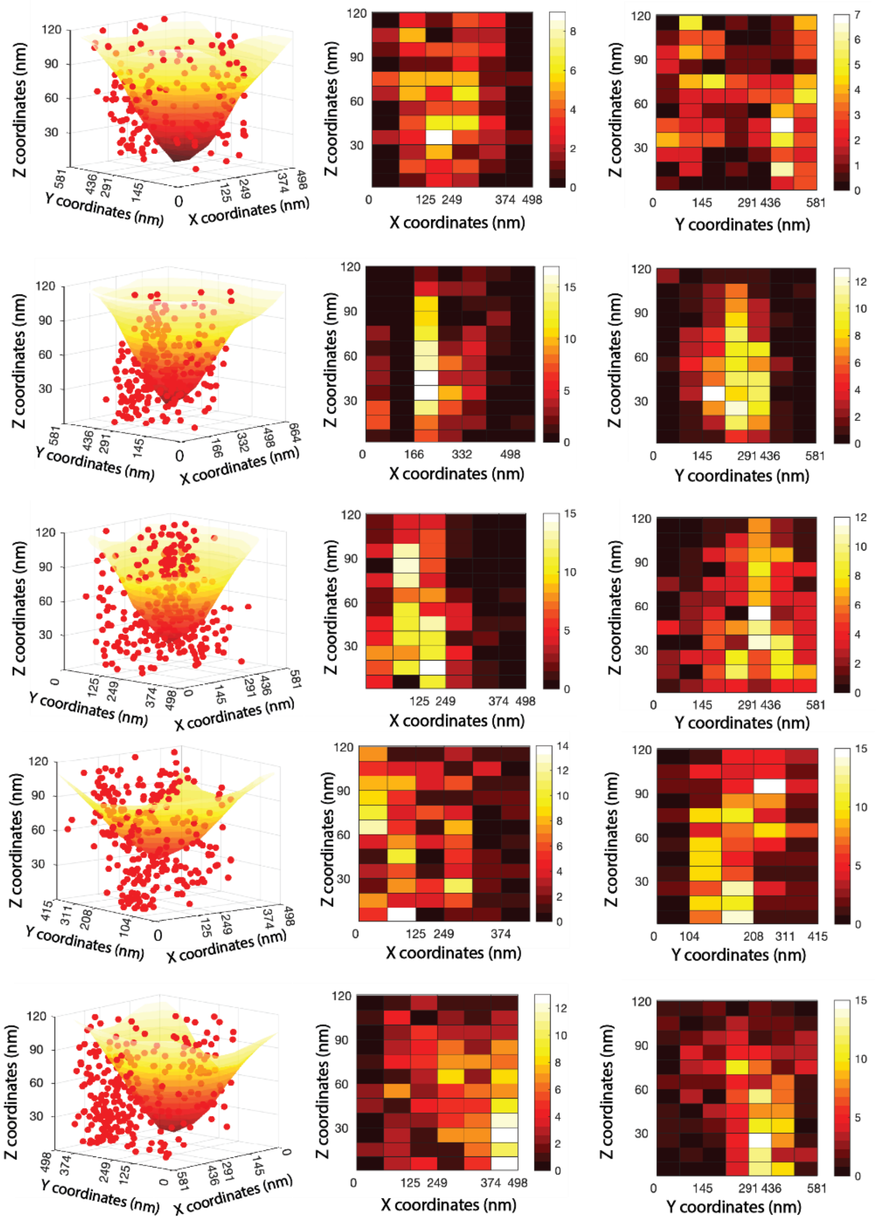

Figure S19. Additional examples of 3D-SMLM localizations of PSGL-1 on vertically oriented microvilli in KG1a cells under shear flow.

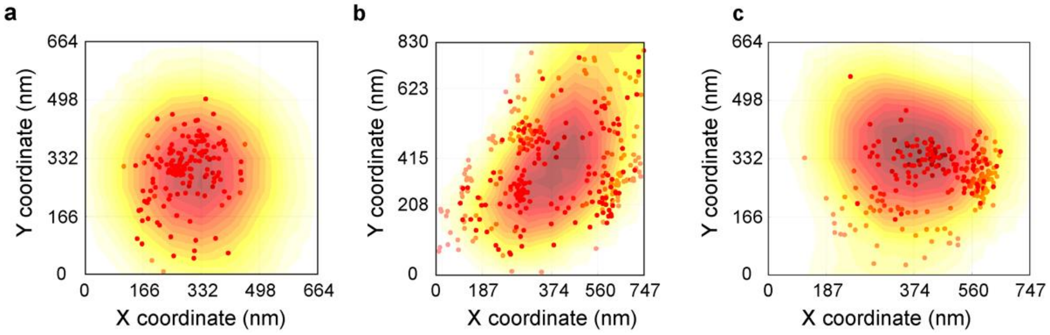

Figure S20. Spatial distribution of adhesion molecules on microvilli on the rolled cells projected on the XY plane. XY projections of 3D single molecule localizations of (a) F-actin, (b) CD44, and (c) PSGL-1 on KG1a cells rolled over E-selectin determined by 3D-SMLM overlaid with a 3D cell surface topography image acquired by TIRFM with MemBrite FX640 stain. The actin, CD44, and PSGL-1 were labeled by AF-488-conjugated phalloidin (actin) or immunolabeled with AF-488-conjugated antibodies (CD44 and PSGL-1). (a), (b) and (c) are the XY projections of the 3D images shown in Figure 7b, 7e, and 7h.

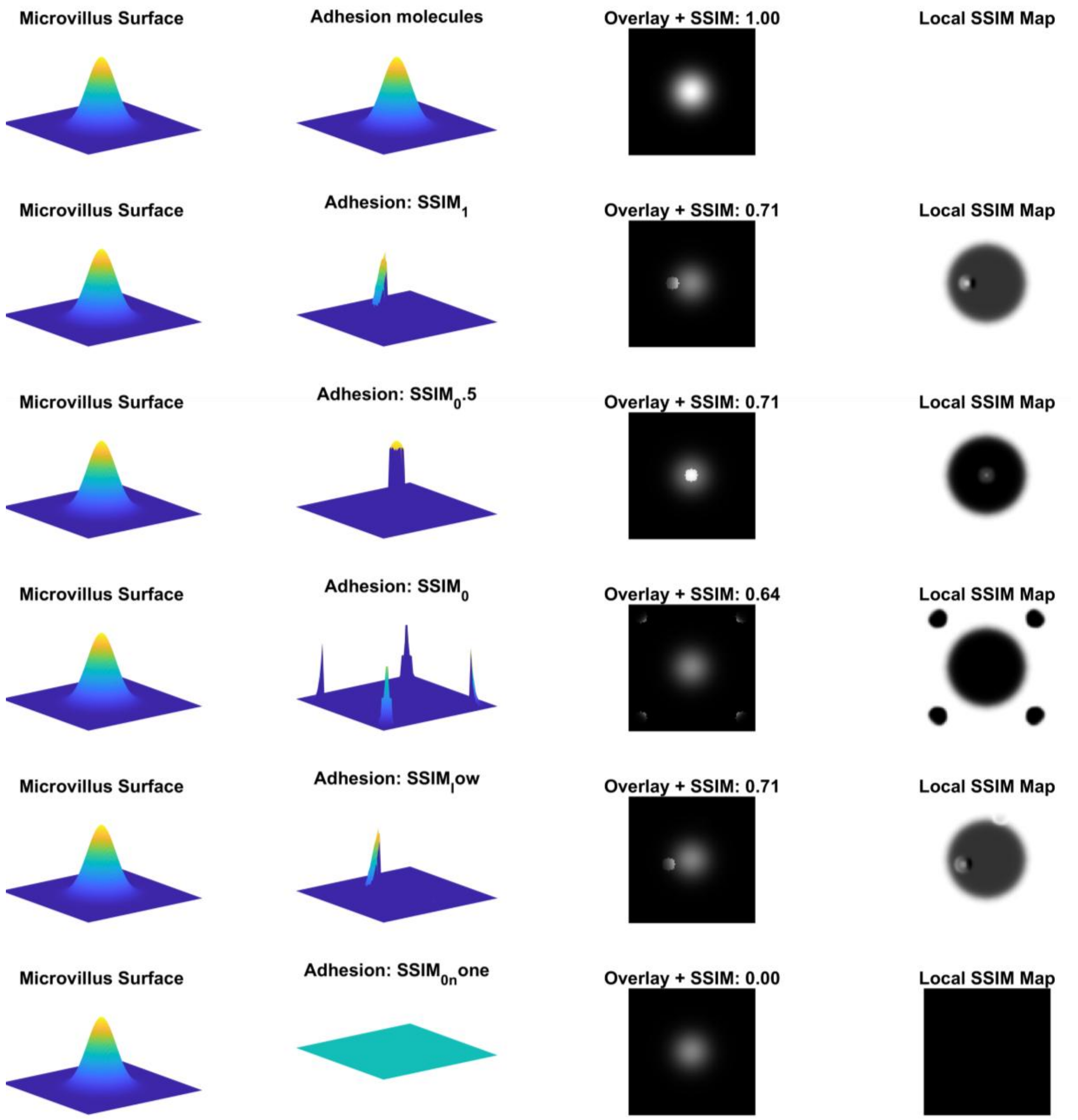

Figure S21. Simulation analysis demonstrating the limited sensitivity of the Structural Similarity Index (SSIM) to localization displacement along the microvillus surface. Simulated surface maps were generated to compare the effects of controlled positional shifts in molecular localizations relative to an idealized microvillus topography.

References

(1) Ghosh, S.; Di Bartolo, V.; Tubul, L.; Shimoni, E.; Kartvelishvily, E.; Dadosh, T.; Feigelson, S. W.; Alon, R.; Alcover, A.; Haran, G. ERM-dependent assembly of T cell receptor signaling and co-stimulatory molecules on microvilli prior to activation. Cell reports 2020, 30 (10), 3434-3447. e3436.

(2) Jung, Y. M.; Riven, I.; Feigelson, S. W.; Kartvelishvily, E.; Tohya, K.; Miyasaka, M.; Alon, R.; Haran, G. Three-dimensional localization of T-cell receptors in relation to microvilli using a combination of superresolution microscopies. Proc. Natl. Acad. Sci. U. S. A. 2016, 113 (40), E5916-E5924. DOI: 10.1073/pnas.1605399113.

(3) Fish, K. N. Total internal reflection fluorescence (TIRF) microscopy. Curr. Protoc. Cytometry 2009, 50 (1), 12.18.11-12.18.13, Review. DOI: 10.1002/0471142956.cy1218s50 Scopus.
